# Exploring the Relationship Between Dolutegravir and Diabetes Using Routine Data of Patients on Anti-Retroviral Treatment in a High HIV Prevalence Setting

**DOI:** 10.18103/mra.v14i2.7309

**Published:** 2026-02-28

**Authors:** Samson Haumba, Thokozani Maseko, Fezokuhle Khumalo, Nakekelo Mndzebele, Clara Nyapokoto, Sylvia Ojoo

**Affiliations:** 1 Center for Global Health Practice and Impact, Georgetown University, Mbabane, Eswatini; 2 Center for Global Health Practice and Impact, Georgetown University Medical Center, Washington DC, USA; 3 Ministry of Health, Mbabane, Eswatini

**Keywords:** Type 2 Diabetes Mellitus, People living with HIV, antiretroviral treatment, dolutegravir-based regimens, high HIV prevalence setting

## Abstract

**Background:**

Dolutegravir has been associated with excessive weight gain and type 2 diabetes mellitus (T2DM) The study aimed to describe the incidence and prevalence of type 2 diabetes mellitus in a cohort of patients exposed and non-exposed to dolutegravir containing antiretroviral therapy in Eswatini.

**Methods:**

We conducted a retrospective observational study using routinely collected data from electronic medical records for adults aged ≥25years active on antiretroviral therapy in two of the country’s regions between 01 October 2018 and 31 August 2025. Included patients were adults (≥25 years) active on antiretroviral therapy with no prior diagnosis of diabetes mellitus. The primary outcome was a documented diagnosis of type 2 diabetes mellitus among dolutegravir initiators- patients who started antiretroviral therapy on a dolutegravir-based regimen; dolutegravir transitioners- patients previously on a non-dolutegravir regimen who transitioned to a dolutegravir-based regimen; and non- dolutegravir users- patients that were never exposed to dolutegravir. Multiple imputation was used to handle missing data in body mass index and antiretroviral therapy duration. Descriptive statistics were used to describe patient characteristics, and Poisson log link regression was used to compute incidence risk ratios.

**Results:**

A total of 102330 patients were analyzed (21938 in dolutegravir initiators group, 73967 in dolutegravir transitioners group and 6425 in non-dolutegravir users group). The participant mean age was 43.7±11.7years, the mean duration on antiretroviral therapy was 9.4±3.9years and mean body mass index (BMI) was 27.5±6.2 kg/m^2^. The overall prevalence of type 2 diabetes was 2.0% (1.2% among dolutegravir initiators; 2.3% among dolutegravir transitioners and 1.4% among non-dolutegravir users) and the overall incidence rate was 3.6 cases per 1000person-years [Non-dolutegravir Users-6.2 per 1000person-years), Dolutegravir Transitioners (3.6 per 1000person-years) and Dolutegravir Initiators (2.9 per 1000person-years)]. After adjusting for age, BMI, and duration on antiretroviral therapy, Dolutegravir Initiators (aIRR=0.4) and Dolutegravir Transitioners (aIRR=0.4) had a lower risk than Non-Dolutegravir Users.

**Conclusion:**

There was a protective relationship for developing type 2 diabetes mellitus for patients on dolutegravir-based regimens in comparison to non-dolutegravir regimens. All patients commencing antiretroviral therapy should have baseline and ongoing blood glucose monitoring as a critical component in the management of HIV.

## Introduction

Type 2 diabetes mellitus (T2DM) cases among people living with HIV (PLHIV) have been a growing concern since early 2000, typically because of two major factors: the extended life expectancy of PLHIV due to effective antiretroviral therapy (ART) and the metabolic side effects associated with some ART medications^1^. A positive relationship between HIV and T2DM was reported as early as 2007 [r=0.346, p=0.004]^2^ and 2010 [r=0.440, p<0.001].^3^ Through this pathway, HIV infection could lead to the development of T2DM by triggering a pro-inflammatory state secondary to cell apoptosis initiated by HIV glycoprotein 41 (gp41) and glycoprotein 120 gp120 leading to elevated inflammatory cytokines such as tumor necrosis factor alpha (TNF-α), interleukins, and C-reactive protein (CRP) levels.^4^ This elevation in inflammatory cytokines is associated with impaired insulin action in skeletal muscles,^5^ and suppression of the production of adiponectin, an adipose-specific collagen-like molecule^6^ that has been observed to have antidiabetic, anti-atherosclerotic and anti-inflammatory functions, leading to impaired insulin sensitivity,^6^ and the eventual development of T2DM. HIV related inflammatory markers are also causally associated with the premature onset of chronic diseases,^7^ particularly T2DM.^8^

On the other hand, there are celebrated successes of ART of the longevity of PLHIV on ART but this also contributes to increased incidence of aging chronic diseases such as T2DM among PLHIV.^9–11^ It has been postulated that ageing is accompanied by a loss of muscle mass, an increase in body fat, leading to insulin resistance,^12–15^ metabolic syndrome, and T2DM. The traditional risk factors for T2DM, sex and body mass index (BMI) also occur among PLHIV on ART. Being overweight or obese increased the odds of having T2DM.^10,11^

Dolutegravir (DTG), an integrase strand transfer inhibitor, is recommended by the World Health Organization (WHO) as the preferred antiretroviral therapy option for all population groups since 2018.^16^ In Eswatini, DTG was adopted and implemented to scale since October 2018, with programmatic data showing that over 94% of people living with HIV on ART are now receiving DTG-based regimens. Findings from qualitative studies showed that PLHIV on ART experience weight gain post ART initiation and with DTG-based regimens^17^ with documented increased appetite, overeating, and consequently weight gain.^18^ Weight gain among patients on DTG has been reported to be excessive compared to other ART regimens,^19–23^ although as it has also been reported in PLHIV on non-nucleoside reverse transcriptase inhibitors (NNRTIs), nucleoside reverse transcriptase inhibitors (NRTIs) and protease inhibitors (PIs).^24–26^ Case studies that reported diabetes among PLHIV on DTG have shown that all the cases were above the age of 40 years;^12–14^ observational studies suggest that being on ART for longer than 3 years, regardless of the regimen, was a predictor of T2DM^10,28^ and long-term use of ART is a predictor of metabolic syndrome, ^29–32^ a precursor for T2DM.

However, since its introduction, some health providers perceive DTG as a key driver in the development of T2DM among PLHIV on ART in the recent years despite many alternative documented pathways and predictors of T2DM among PLHIV.^18^ There are reports of PLHIV developing T2DM within 12 months of DTG and sometimes as early as 2 months post DTG initiation.^12–14^ In one study, 16.4% patients were switched back to a non-DTG-based regimen because they developed T2DM.^28^ Conversely, some case studies report that T2DM among PLHIV on DTG only happened among those above the age 40 years.^12–14^ Among the ART experienced, some patients transitioned to DTG could have been already pre-diabetic at initiation.^12,13^ On the contrary, a protective relationship between DTG and T2DM has been reported (OR<1).^10,28^ This suggests that there could be other factors that predispose PLHIV on ART to develop T2DM other than DTG.

This study is significant because while it has been shown that people living with HIV have a higher incidence of diabetes mellitus (DM) relative to the general population, there are conflicting reports about the role of newer antiretroviral like DTG on T2DM. This study aimed to explore the relationship between the incidence of T2DM among DTG-exposed and non-DTG-exposed adults living with HIV. In addition, the study explored the risk factors associated with T2DM among adults on ART. The study will reinforce evidence-based management practices to inform switching from a DTG-based regimen to a non-DTG regimen due to hyperglycaemia for patients on ART as well as the need for routine monitoring of blood sugar for patients on ART.

## Methods

### STUDY DESIGN AND SETTING

A retrospective cohort study conducted on patients on ART between October 1 2018 and August 31 2025 was conducted in a setting of high HIV prevalence that has achieved UNAIDS HIV epidemic control targets of 95-95-95. All PLHIV aged 25years and above receiving HIV care in 85 health facilities providing HIV treatment services in two out of four regions in the country were included In 2018, the country adopted DTG based regimens, and patients starting on ART received Tenofovir/lamivudine/dolutegravir (TLD) except those with contraindications, while patients already on ART, on a standard first or second line and not failing on their current regimen, were transitioned to a DTG based regimen, with most patients on first line ART regimen transitioned to TLD. Healthcare workers were trained on the DTG transition process and the HIV guidelines that guide the implementation of DTG based ART regimens in Eswatini.

Patients were drawn from all health facility types including 5 hospitals (with in-patient services and has medical officers onsite), 1 rural health center (equivalent to a multi-department health center and has medical officers onsite), 5 public health units (a nurse-led maternal and child welfare clinic), and 74 primary health clinics (providing nurse-led clinical services).

### STUDY PATIENTS, SAMPLE SIZE AND SAMPLING

**Figure 1:**
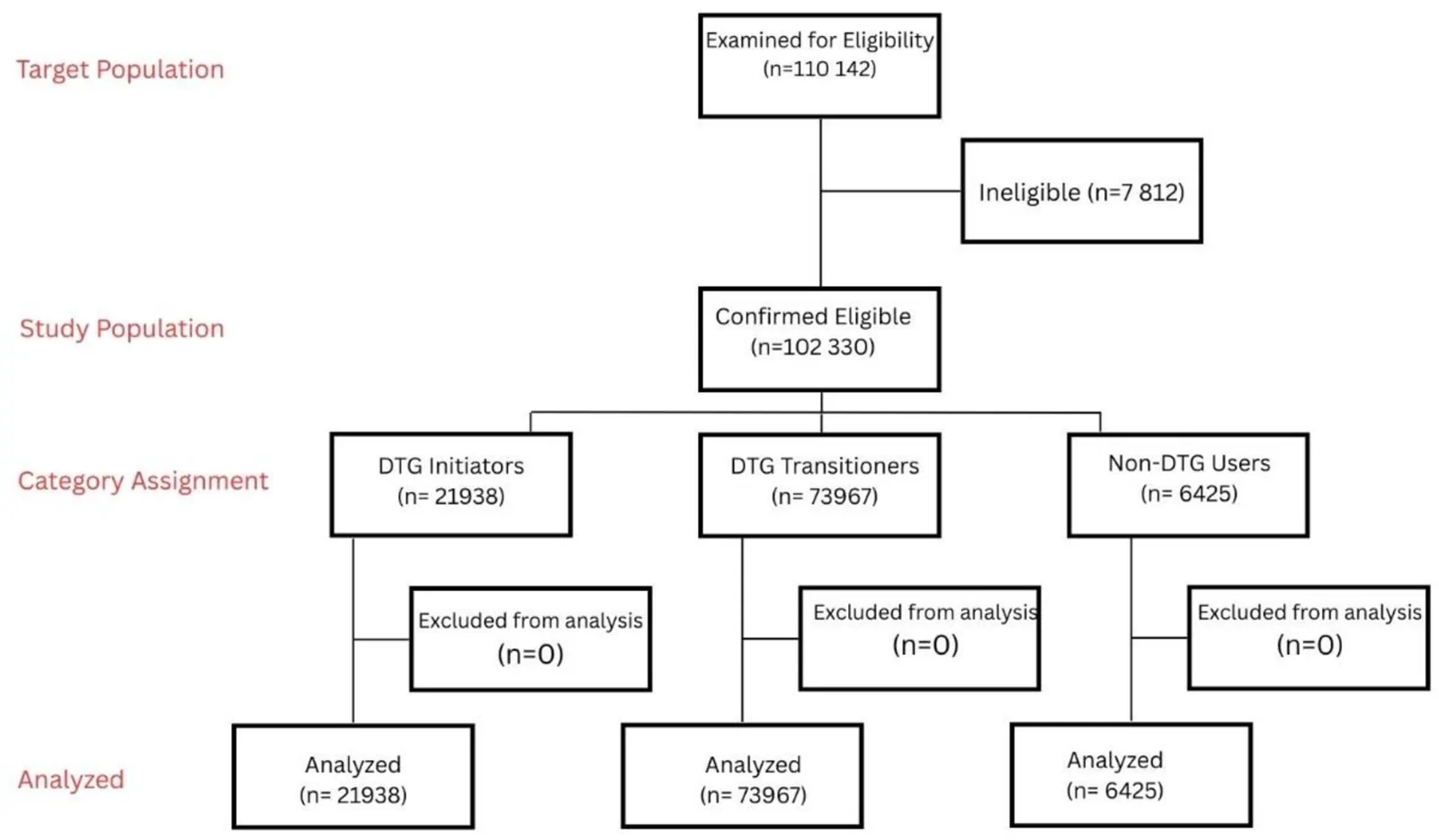
Flowchart of the study population

We abstracted data for the study from the electronic medical records called the Client Management Information System (CMIS) documenting client details and clinical services received for all patients who were active on ART on 01 October 2018 or were initiated on ART between 01 October 2018 and 30 September 2024 and all were followed until 31 August 2025. All patient data were de-identified by removing direct identifiers and replacing them with research-specific identifiers to ensure anonymity. Data were retrospectively analyzed. A total of 77134 PLHIV were active on ART on 01 October 2018, and 33008 initiated on ART between 01 October 2018 and 31 August 2024, making the total study population 110142. We excluded 7790 patients who were below 25years old, and 22 patients who had been diagnosed with diabetes before the age 25years (type 1 diabetes), hence 7812 patients were excluded. The total eligible patients were 102330 patients (Figure 1) and categorized as follows: DTG Initiators (ART-Naïve Baseline)-ART-naïve individuals who commenced first-line therapy with a DTG-based regimen; DTG Transitioners (ART-Experienced)-, Individuals previously receiving non-DTG ART who transitioned to a DTG-based regimen; Non-DTG Users (DTG-Unexposed)-Individuals on non-DTG ART regimens with no prior exposure to DTG.

The key variables included ART start date, T2DM diagnosis date, ART regimen, ART transition date, sociodemographic and clinical information (Table 1). Duration on ART was categorized into 6 categories using 3year bands from ≤3 to >15years. Age was grouped into 3 categories using 15-year age bands, that is, 2540 years, 41-55 years and >55 years. ^12–14^ Patients’ BMI at ART initiation was grouped into 4 groups from being underweight (BMI<18.5), normal weight, overweight and obese.^33^ Since the study aimed to describe if exposure to DTG influenced T2DM development, patients were stratified into three groups, that is, Initiated on DTG, Transitioned to DTG, and Never been on DTG. Type 2 diabetes was determined as documented diabetes diagnosis and prescription of diabetes medication.^34^ We used the International Classification of Diseases 10^th^ revision (ICD-10-CM) codes for type 2 diabetes with the first letter followed by two digits and did not further specify the categories based on complications and type of medications used to treat the T2DM.

### STATISTICAL ANALYSIS

The data was extracted in.csv format and imported into STATA 19.5 (College Station, TX) for cleaning and analysis. Multiple imputation using multivariate normal (MVN) in mlong style constrained within normal limits was used to impute missing at random values in BMI and duration on ART which had a monotone missing pattern. Bayesian iterative Markov chain Monte Carlo (MCMC) convergence was achieved in 71st iteration, and graphical presentation of autocorrelation (Annex 1)and worst linear function plot (Annex 2)showed no autocorrelation of the worst linear function. Proportions described categorical variables, while distribution of continuous variables was assessed through QQ-plot and mean with standard deviation described parametric continuous variables and median with interquartile range described non-parametric continuous variables. Descriptive analysis summarized key patient characteristics, presented in Table 2, disaggregated by T2DM status and count data were presented as percentages [n, (%)]. The outcome variable, T2DM status, had no missing observations. To ensure accuracy in predictive analysis, “mi split” command was used to include non-missing values in the analysis. Person-time was computed based on duration on ART of patients. Crude incidence rates (defined as the number of new T2DM cases in the population of patients on ART) divided by person-time were computed and reported per 1000person-years. To compute the incidence risk of developing diabetes in the overall ART sample, regardless of stratification group, unadjusted risk-ratios were computed using bivariable Poisson regression. In all cases, a p<0.05 was considered statistically significant. Variables with a significance level of p<0.2 in bivariable analysis were included in the multivariate analysis. The relative risk of developing T2DM was computed using multivariable Poisson regression analysis with log link function and vce robust option, and incidence risk ratio (IRR) was reported. We assessed quality of the final adjusted estimated model using chi-squared goodness-of-test ^35^, the model adequately fitted the data (P>0.05. Overdispersion was assessed using “overdisp command” ^36^ and the “prcounts prpoisson” and poisgof” commands ^37,38^, and there was no over dispersion (P > |t|= 0.103). The same analysis steps were taken to compute the incidence risk per stratification group.

## Results

### DESCRIPTION OF STUDY POPULATION

This study analysed 102330 patients, the majority being female (n=65498, 64.0%) (Table1). The mean age was 43.7±11.7 and the majority were single (n=62776, 62.1%) . The mean BMI at ART initiation was 27.5±6.2 kg/m^2^, and BMI varied from obese ( n=58112, 56.8%), overweight (n=19203, 18.8%) normal BMI (n=23422, 22.7%) . The mean duration on ART in this study population was 9.4±3.9 years, but the patients with T2DM had a higher mean duration (10.9±3.8 years) compared to those without T2DM (9.4±3.9 years).

### Prevalence of type 2 Diabetes and associated clinical and demographic factors

Overall, T2DM was reported in 2.0% (n=2056) of the study participants, with the highest prevalence among those who were DTG Transitioners at 2.13% (n=1713), Non-DTG Users at 1.4%, (n=90), and least DTG Initiators at 1.2% (n=253). A regional difference in T2DM prevalence was noted, being higher in patients in Manzini than in Lubombo 2.2%, (n=1045) vs 1.7% (n=651). Patients aged 56 years and above had the highest prevalence of T2DM at 6.6% (n=1020) among the age categories, males had a higher prevalence than females 2.4%, (n=870) vs 1.8%, (n=186). Patients that were either divorced/separated/widow also had the highest prevalence of T2DM, 5.0% (n=156) among marital status categories. In addition, the mean duration on a DTG regimen was 41.7±22.2 months and was higher among patients with T2DM (43.1±21.7) compared to those without T2DM (41.7±22.2 months). At the end of the study, most of the patients were on a DTG based regimen 93.7% (n=95905), with DTG Initiators constituting 21.4% (n=21938), while the DTG Transitioners constituted 72.3% (n=73967). Most of the patients (99.6%, n=101895) were on a non-PI ART regimen.

The patients with T2DM had a higher mean BMI (29.4±6.4 kg/m^2^) compared to the non-diabetic (27.5±6.2 kg/m^2^). The prevalence of T2DM increased with increasing BMI, with the highest prevalence among those who were mildly obese at 4.0% (n=482). The prevalence of T2DM increased with increasing duration on ART. Patients who have been on ART for more than 15 years had a prevalence of 4.0% (n=352) while those with a duration of less than 15 years had a T2DM prevalence of 2.6% (n=514). Among the DTG Initiators, the T2DM prevalence increased from 1.9% (n=879) among those who had been on DTG for 3 years or less, to 2.2% (n=81) in those on DTG for more than 6 years (Table 2).

### INCIDENCE OF TYPE 2 DIABETES MELLITUS

As shown in table 3, the overall incidence of T2DM was 3.6 cases per 1000person-years (PY) [3.4-3.7], with the highest incidence among Non-DTG Users (6.2 [5.1-7.7]), followed by DTG Transitioners (3.6 [3.5-3.8]), and DTG Initiators had the lowest incidence (2.9 [2.6-3.3]). The incidence was higher among females compared to males (4.3 per 1000 PY vs 3.2 per 1000 PY), and these findings were consistent when stratified by ART experience. Incidence increased with age, peaking in individuals >55 years, who had 11.1 per 1000 PY [10.5–11.9] cases per 1000 PY overall, and up to 21.7 per 1000 PY [16.3–28.8] among Non-DTG Users. Incidence was notably high among widowed/ separated/divorced patients (8.6 per 1000 PY [7.3–10.0]**)**, in particular Non-DTG Users (24.8 per 1000 PY [12.4–49.5]). Incidence also increased with increasing BMI, with obese patients having the highest overall incidence rate of 6.4 per 1000 PY [6.0– 6.9].

The overall incidence rate was high within the first 3 years on ART and more than 15 years on ART (6.7 per 1000 PY vs 6.7 per 1000 PY), with Non-DTG Users consistently having a high incidence across all three strata. Among the patients on DTG, incidence was similar within all year durations as the incidence within the first 3 years was 3.4 per 1000 PY [3.2–3.7], increasing to 3.6 per 1000 PY [3.7– 3.8] after 4–6 years, and slightly declining to 3.4 per 1000 PY [2.7- 4.2] after 6 years. Patients on PI-based regimens experienced much higher incidence (6.1 per 1000 PY [11.7- 22.2]) compared with those on non-PI regimens (3.5 per 1000 PY [2.4– 2.7]). When compared to patients who are currently on non-PI based regimens, incidence was higher in patients on PI-based regimens among DTG Initiators and Non-DTG Users except DTG Transitioners (2.1 per 1000 PY [0.3- 15.2] vs 3.6 per 1000 PY [3.5- 3.8]). The incidence rate was similar between patients whose current facility is a clinic (3.5 per 1000 PY [3.4–3.7]) and hospital (4.0 per 1000 PY [3.5–4.6]), with patients currently using hospital facilities and were Non-DTG Users having the highest incidence (13.0 per 1000 PY [9.1-18.4]).

Several factors were associated with the development of T2DM among ART patients. Females had 30% (aIRR=0.7 [0.6-0.8], p<0.001) lower risk of developing type 2 diabetes compared to males. Widowed, separated, or divorced patients had a 1.3([1.1-1.4], p<0.001) times higher adjusted risk of T2DM compared to singles, while married patients showed a similar adjusted risk (aIRR=1.3 [1.0–1.5], p=0.026). For every one-year increase in age, the risk of developing T2DM increased by 5% (aIRR=1.054 [1.052-1.058], p<0.001). For every one-unit increase in BMI, the risk of developing T2DM increased by 8% (aIRR=1.08 [1.07-1.08], p<0.001).

Similar to age and BMI, for every additional year on ART, the risk of developing T2DM increased by 4% (aIRR=1.04 [1.02-1.06], p<0.001). DTG Initiators had 60% (aIRR=0.4 [0.3-0.6], p<0.001) lower risk of developing T2DM compared to Non-DTG Users. Similarly, DTG Transitioners had 60% (aIRR=0.4 [0.30.6], p<0.001) lower risk of developing T2DM compared to Non-DTG Users. Currently being on a PI-based regimen significantly increased the risk of developing T2DM by 1.9 ([1.2- 2.9], p=0.005) times. Current facility was not a significant factor associated with the development of T2DM (p>0.05). (Table 4).

For the patients that were DTG Initiators; females had 50% (aIRR=0.5 [0.4-0.7], p<0.001) lower risk of developing type 2 diabetes compared to males. Married patients had a 1.6([1.2-2.2], p=0.003) times higher adjusted risk of T2DM compared to patients who were not married, while widowed, separated, divorced patients did not show a significant (p>0.05) association with developing T2DM. For every one-year increase in age, the risk of developing T2DM increased by 6% (aIRR=1.06 [1.05-1.07], p<0.001). For every one-unit increase in BMI, the risk of developing T2DM increased by 9% (aIRR=1.09 [1.07-1.11], p<0.001 Patients who are currently on a PI-based regimen had 5.6 (aIRR=5.6 [2.4-13.2], p<0.001) times the risk of developing T2DM compared to those on a non-PI based regimen. Duration on ART, duration on DTG and Current facility were not significant factors associated with developing T2DM amongst this stratum (p>0.05).

For the patients that were DTG Transitioners; females had 30% (aIRR=0.7 [0.6-0.8], p<0.001) lower risk of developing type 2 diabetes compared to males. Married patients had 1.2([1.1-1.3], p=0.003) times higher adjusted risk of T2DM compared to singles, while widowed, separated, divorced patients did not show a significant (p>0.05) association with developing T2DM.

For every one-year increase in age, the risk of developing T2DM increased by 5% (aIRR=1.053 [1.050-1.056], p<0.001). For every one-unit increase in BMI, the risk of developing T2DM increased by 8% (aIRR=1.08 [1.07-1.09], p<0.001). Similar to age and BMI, for every one-year increase in duration on ART, the risk of developing T2DM increased by 5% (aIRR=1.05 [1.03-1.07], p<0.001). Duration on DTG, whether current ART regimen has a PI and Current facility were not significant factors associated with developing T2DM amongst this stratum (p>0.05).

For the patients that were Non-DTG Users; females had a50% (aIRR=0.5 [0.3-0.9], p=0.011) lower risk of developing type 2 diabetes compared to males. Married patients had 2.0([1.8-3.2], p=0.046) times higher adjusted risk of T2DM compared to unmarried patients, while widowed, separated, divorced patients did not show a significant (p>0.05) association with developing T2DM. For every one-year increase in age, the risk of developing T2DM increased by 3% (aIRR=1.03 [1.011.06], p=0.003). For every one-unit increase in BMI, the risk of developing T2DM increased by 6% (aIRR=1.06 [1.02-1.09], p<0.001). Patients who are currently on a PI-based regimen had 1.7 (aIRR=1.7 [1.0-3.0], p=0.047) times the risk of developing T2DM compared to those on a non-PI based regimen.

## Discussion

The study aimed to explore the relationship between DTG and T2DM through the investigation of prevalence and the incidence of type 2 diabetes mellitus in a cohort of patients living with HIV who were either initiated or transitioned to DTG in comparison to patients who have never received DTG containing antiretroviral therapy. The study found that the overall prevalence of T2DM was 2.0% and incidence rate of T2DM was 3.6 cases per 1000 person-years (PY) in the cohort. The prevalence rate of 2% in this study appears to be lower than the estimated prevalence of 3.6% in the general population from the International Diabetes Federation ^39^ Our study used clinical data from medical records of diagnosed cases rather than universal blood glucose screening, and hence a possibility under-reporting of undiagnosed hyperglycemia.^40,41^ The study highlights a high baseline BMI in this cohort, with 56.8% being obese and 18.8% overweight, among PLHIV. This underscores the need for integrating interventions that address risk factors for non-communicable diseases (NCDs) among PLHIV. ^42,43^

The results of the study highlight a potential protective association of DTG-based regimens against T2DM development. Both DTG Initiators and DTG Transitioners demonstrated a significant 60% lower adjusted risk of developing T2DM compared to the Non-DTG users. This aligns with emerging evidence suggesting that while integrase strand transfer inhibitors (INSTIs) are associated with weight gain, they may not inherently increase the risk of clinical diabetes as severely as older antiretroviral classes.^44,45^ The Non-DTG Users had the highest crude incidence rate of 6.2 per 1000 PY, likely reflecting the metabolic risk of older NRTIs and NNRTIs known for mitochondrial toxicity and adipocyte dysfunction.^20,46^ In contrast, the lower incidence in DTG Initiators 2.9 per 1000 PY could suggest that early initiation on DTG regimen may reduce the systemic inflammation that is associated with metabolic dysregulation.^47,48^

The risk of T2DM increased by approximately 5% for every one-year increase in age and by 8% for every one-unit increase in BMI. These findings confirm that as the PLHIV population in Eswatini ages, their risk profile converges with that of the general population.^49,50^ The significant association between obesity at ART initiation and subsequent T2DM incidence (6.4 per 1000 PY) highlights the need for aggressive weight management early in the treatment course.^34,51^ This finding implies that clinical management must aggressively target obesity and aging-related comorbidities among PLHIV.^52,53^ Regardless of the specific antiretroviral regimen, it is evident that traditional risk factors such as age and BMI remain the primary risk factors of T2DM.

While females had a higher crude incidence rate compared to males, 4.3 vs. 3.2 per 1000 PY, the females demonstrated a 30% to 50% lower adjusted risk of T2DM compared to males after controlling for age and BMI. This suggests that the higher absolute number of T2DM cases female patients in the study is largely driven by a higher baseline BMI and different fat distribution patterns rather than an inherent biological susceptibility to ART-induced hyperglycemia.^54,55^ In contrast, males in this cohort appeared more vulnerable to T2DM at lower BMI thresholds, which aligns with findings from other studies that visceral adiposity in men may be more metabolically active and inflammatory.^56,57^ These results necessitate sex-disaggregated metabolic screening protocols ^58^ within Eswatini’s HIV clinics.

Protease Inhibitors users experienced a nearly two-fold increased risk of T2DM (aIRR = 1.9) highlighting established metabolic risks associated with PIs. This risk was even more pronounced among DTG Initiators who are currently on a PI based regimen (aIRR = 5.6), confirming that PIs are potent drivers of insulin resistance and dyslipidemia.^2,3^

Our analysis revealed that the risk of T2DM increased by 4% for every additional year spent on ART. This finding could indicate the cumulative impact of chronic HIV infection and long-term exposure to various ART regimens.^59,60^ In addition, the study demonstrated that those who transitioned to DTG maintained a higher prevalence of T2DM (2.3%) compared to the DTG Initiators (1.2%) which is consistent with the observation that transitioning may not immediately erase the metabolic damage accrued during years of treatment with older- regimens.^10,59^

Although our findings indicate that dolutegravir (DTG) use is protective against the development of type 2 diabetes mellitus (T2DM), this protective effect may not be uniform across all clinical contexts. For example, in one study there was non-statistically significant difference in the incidence of hyperglycaemia in the exposed group compared to control group, while in another showed that hyperglycaemia was associated with DTG^65,68^. In addition, evidence from a cross-sectional study conducted in Zimbabwe demonstrated that people living with HIV who were co-infected with tuberculosis (TB) and receiving a double dose of DTG as part of rifampicin-based TB treatment had a higher risk of hyperglycaemia and impaired glucose regulation, both of which are precursors to T2DM.^64^ This increased metabolic risk is thought to be related to pharmacokinetic interactions between DTG and rifampicin, which necessitate DTG dose escalation and may amplify its effects on glucose metabolism.^16,44^ These findings suggest that while standard-dose DTG may confer metabolic protection among the broader population of people living with HIV, individuals with HIV/TB co-infection represent a clinically distinct subgroup at elevated risk for dysglycaemia. Some studies have also suggested that despite a trend towards an initial increase in insulin resistance that maybe associated with DTG, and potentially hyperglycaemia through week 12 of initiation, this followed by a decline through week 36 and at 48 weeks and were not significantly different from baseline^65^. Consequently, routine screening for glucose abnormalities among people living with HIV and TB is warranted to facilitate early detection and management of impaired glucose regulation, thereby reducing progression to overt diabetes mellitus.^43^ Integrating regular glycaemic monitoring into HIV/TB co-management protocols may help mitigate emerging non-communicable disease risks while preserving the virologic benefits of DTG-based regimens.

Our findings may have implications on our understanding of the relationship between DTG and T2DM as well as clinical practice. For instance, patients initiated on DTG and those who transitioned from other regimens to DTG showed a significantly lower adjusted risk of developing T2DM compared to those who had never been on DTG. This demonstrates the role of other factors such as age, BMI, and duration on ART on the risk of T2DM among patients on ART. From a clinical management perspective, our study reinforces the need for continuous metabolic monitoring among patients on ART, regardless of ART regimen. It is imperative that for proper clinical management of all patients initiating ART a baseline test for fasting blood sugar should be conducted and documented and that switching regimen may not always be the answer in cases of hyperglycaemia^65,66^. Furthermore, the high incidence rates within the first three years of ART (6.7 per 1000 PY) suggest that metabolic monitoring should begin immediately upon treatment initiation, particularly for patients with a high baseline BMI. ^14,61^ In addition, there should be ongoing screening for T2DM particularly in older patients, patients with high BMI, history of exposure to Protease Inhibitors or considerable duration on ART, irrespective of exposure to DTG and patients with increased metabolic risks- such as those co-infected with Tuberculosis and on antituberculosis medication. Our study used routine data to draw conclusions on the relationship between DTG and T2DM, and that could have drawbacks. We recommend prospective randomized controlled studies to investigate this relationship further. We also recommend that the practice of switching patients from DTG based regimen to a non-DTG based regimen should be based on evidence that DTG is responsible for the genesis of the hyperglycaemia other than the other known risk factors. Furthermore, our findings imply the need for the integration of NCD screening into Eswatini’s national HIV program.

To sustain the gains made in HIV survival, Eswatini must move toward a differentiated care model that includes regular BMI tracking, and glucose screening as standard components of the ART visit.^14,62,63^

### Strengths and Limitations

The use of routine data in this study has several limitations. Data incompleteness/missing data stands out as the biggest limitation. Some patients were excluded from the analysis because of missing key variables. However, through the multiple imputations, we were able to mitigate the impact of missing data. The study estimated the prevalence of T2DM based on diagnosed cases as captured in the electronic medical records which may have a limitation of excluding undiagnosed cases. The estimated prevalence might have been understated. Additionally, baseline blood glucose at the time of transition to DTG was not consistently reported, possibly underestimating T2DM in the DTG Transitioners. Despite these limitations, this study is adding to the body of knowledge on the relationship between DTG and T2DM.

## Figures and Tables

**Table 1:** Demographic and Clinical Variables of the Study Population disaggregated by their Type 2 Diabetes Mellitus status

	Type 2 Diabetes Mellitus		Total number of patients
	Yes	No	
**Overall (N)**	**2056 (2.0%)**	**100274(98.0%)**	**102330 (100%)**
**Region of treatment initiation**			
Manzini	1405 (2.2)	63356 (97.8)	64761 (63.3)
Lubombo	651 (1.7)	36918 (98.3)	37569 (36.7)
**Age (years)**			
Mean ±SD	55.8±11.1	43.5±11.6	43.7±11.7
25 – 40 years	112 (0.3)	40767 (99.7)	40879 (40.0)
41-55 years	924 (2.0)	45070(98.0)	45994(45.0)
≥ 56 years	1020 (6.6)	14437 (93.4)	15457 (15.0)
**Sex**			
Female	1186 (1.8)	64312 (98.2)	65498 (64.0)
Male	870 (2.4)	35962 (97.6)	36832 (36.0)
**Marital Status**			
Divorced/Separated/Widow	156 (5.0)	2960 (95.0)	3116 (3.1)
Married	1084 (3.1)	34052 (96.9)	35136(34.8)
Single (Never married)	808 (1.3)	61968 (98.7)	62776 (62.1)
**BMI at ART initiation (kg/m^2^)**			
Mean ±SD	29.4±6.4	27.5±6.1	27.5±6.2
Underweight	21 (1.2)	1752(98.8)	1773 (1.7)
Normal	317 (1.4)	22925 (98.6)	23242(22.7)
Overweight	567 (3.0)	18636 (97.0)	19203 (18.8)
Obese	1151 (2.0)	56961 (98.0)	58112 (56.8)
**ART experience status**			
Non-DTG Users	90 (1.4)	6335 (98.6)	6425 (6.3)
DTG Initiators	253 (1.2)	21685 (98.8)	21938 (21.4)
DTG Transitioners	1713(2.3)	72254 (97.7)	73967 (72.3)
**Currently ART regimen has a PI-based regimen**			
PI based	37 (8.5)	398 (91.5)	435 (0.4)
Non-PI based	2019 (2.0)	99876(98.0)	101895(99.6)
**Duration on ART (Years)**			
Mean ±SD	10.9±3.8	9.4±3.9	9.4±3.9
≤3	79 (1.1)	7058 (98.9)	7137 (7.2)
4-6	110 (1.1)	9571 (98.9)	9681 (9.8)
7-9	393 (1.5)	25433 (98.5)	25826 (26.0)
10-12	568 (2.0)	27527 (98.0)	28095 (28.3)
13-15	514 (2.6)	19313 (97.4)	19827 (20.0)
>15	352 (4.0)	8298 (96.0)	8650 (8.7)
**Duration on DTG (Months)**			
Mean ±SD	43.1±21.7	41.7±22.2	41.7±22.2
≤3	879 (1.9)	44637 (98.1)	45516 (47.5)
4-6	1006 (2.2)	45724 (97.8)	46730 (48.7)
>6	81 (2.2)	3578 (97.8)	3659 (3.8)

Overall Prevalence of T2DM= 2056/102330 = 2.01%

**Table 2:** Association between Demographic and Clinical variables of the study participants and Type 2 Diabetes Mellitus

Parameter	DTG Initiators n(%)	DTG Transitioners n(%)	Non-DTG Users n(%)	P-value
**Type 2 Diabetes mellitus**	**253(1.2)**	**1713(2.3)**	**90(1.4)**	**<0.001**
**Timing of T2DM diagnosis to ART Exposure**				
Prior to ART exposure	52 (0.2)	74 (0.1)	3.1 (0.1)	**-**
Post ART exposure	201(0.9)	1639(2.2)	86(1.3)	**-**
**Timing of T2DM to DTG exposure**				
Prior to DTG exposure	52(0.2)	126(0.2)	-	**-**
Post DTG exposure	201(0.9)	1513(2.0)	-	**-**
**Region of treatment initiation**				
Manzini	177(1.2)	1201(2.6)	27(0.7)	<0.001
Lubombo	76 (1.0)	512(1.9)	63(2.4)	<0.001
**Age (years)**				
25 – 40 years	29(0.2)	79(0.3)	4(0.1)	0.063
41-55 years	135(1.9)	751(2.0)	38(1.5)	0.097
≥ 56 years	89(6.4)	883(6.7)	48(4.9)	0.078
**Sex**				
Female	111(0.9)	1022(2.1)	53(1.2)	<0.001
Male	142(1.5)	691(2.7)	37(1.9)	<0.001
**Marital Status**				
Divorced/Separated/Widow	15(3.5)	133(5.2)	8(5.5)	0.311
Married	136(2.6)	900(3.2)	48(3.3)	0.101
Single (Never married)	102(0.6)	673(1.6)	33(0.8)	<0.001
**BMI at ART initiation (kg/m^2^)**				
Underweight	2(0.6)	17(1.2)	2(15.4)	<0.001
Normal	34(0.6)	274(1.6)	9(4.4)	<0.001
Overweight	64(1.6)	487(3.3)	16(7.8)	<0.001
Obese	60(2.7)	404(4.1)	18(14.6)	<0.001
**Current ART regimen has a PI-based regimen**				
PI based	4(16.7)	1(1.3)	32(9.5)	0.009
Non-PI based	249(1.1)	1712(2.3)	58(1.0)	<0.001
**Duration on ART (Years)**				
≤3	76(1.1)	1(0.8)	2(10.0)	0.030
4-6	104(1.2)	3(0.5)	3(1.4)	0.354
7-9	35(1.1)	339(1.7)	19(0.7)	<0.001
10-12	-	550(2.1)	18(1.1)	0.005
13-15	-	484(2.6)	30(2.4)	0.629
>15	-	334(4.1)	18(3.2)	0.275
**Duration on DTG (Years)**				
≤3	99(1.2)	780(2.1)	-	<0.001
4-6	144(1.1)	862(2.5)	-	<0.001
>6	10(1.0)	71(2.7)	-	0.001

**Table 3:** Incidence of Type 2 Diabetes Mellitus and by Dolutegravir regimen /ART experience status

Parameter	Overall Incidence Rate [95%CI] per 1000 PY	DTG Initiators Incidence Rate [95%CI] per 1000 PY	DTG Transitioners Incidence Rate [95%CI] per 1000 PY	Non-DTG Users Incidence Rate [95%CI] per 1000 PY
**Person-time**	573309.3	86294.9	472659.9	14354.4
**Overall**	3.6 [3.4-3.7]	2.9 [2.6-3.3]	3.6 [3.5-3.8]	6.2 [5.1-7.7]
**Sex**				
Male	3.2 [3.0-3.4]	2.2 [1.9-2.7]	3.3 [3.1-3.5]	5.0 [3.8-6.6]
Female	4.3 [4.0-4.6]	3.9 [3.2-4.6]	4.3 [4.0-4.6]	9.8 [7.1-13.5]
**Current age (years)**				
25-39	0.5 [0.4-0.6]	0.6 [0.4-0.8]	0.5 [0.4-0.6]	0.6 [0.2-1.7]
40-55	3.4 [3.2-3.6]	4.5 [3.8-5.3]	3.2 [3.0-3.5]	6.4 [4.7-8.8]
>55	11.1 [10.5-11.9]	14.8 [12.0-18.2]	10.6 [10.0-11.4]	21.7 [16.3-28.8]
**Marital status**				
Single	2.3 [2.2-2.5]	1.6 [1.3-1.9]	2.5 [2.3-2.7]	3.4 [2.4-4.7]
Married	5.2 [4.9-5.5]	6.6 [5.6-7.8]	4.9 [4.6-5.2]	13.8 [10.4-18.4]
Widowed, Separated, Divorced	8.6 [7.3-10.0]	9.3 [5.6-15.4]	8.2 [6.9-9.7]	24.8 [12.4-49.5]
**BMI at ART Initiation**				
Underweight	2.0 [1.3-3.0]	1.7 [0.4-6.9]	1.8 [1.1-2.9]	28.9 [7.2-45.4]
Normal	2.3 [2.1-2.6]	1.8 [1.3-2.5]	2.3 [2.1-2.6]	9.3 [4.8-17.9]
Overweight	4.9 [4.5-5.3]	4.4 [3.4-5.6]	4.9 [4.5-5.3]	16.2 [9.9-26.4]
Obese	6.4 [6.0-6.9]	7.1 [5.8-8.7]	6.1 [5.6-6.6]	28.9 [19.7-42.5]
**Duration on ART (years)**				
<=3	6.7 [5.4-8.4]	7.0 [5.6-8.8]	1.3 [0.2-9.4]	16.4 [4.1-65.8]
4-6	2.2 [1.8-2.7]	2.2 [1.9-2.8]	0.9 [0.3-2.7]	5.7 [1.8-17.7]
7-9	2.6 [2.3-2.9]	1.8 [1.3-2.5]	2.7 [2.4-3.0]	4.0 [2.5-6.3]
10-12	3.3 [3.0-3.6]	-	3.2 [3.0-3.5]	4.1 [2.6-6.5]
13-15	4.2 [3.8-4.6]	-	4.1 [3.7-4.4]	9.1 [6.4-13.1]
>15	6.7 [6.0-7.4]	-	6.5 [5.8-7.2]	14.2 [9.0-22.5]
**Duration on DTG (years)**				
<=3	3.4 [3.2-3.7]	6.5 [5.4-7.9]	3.3 [3.0-3.5]	-
4-6	3.6 [3.7-3.8]	2.2 [1.9-2.6]	4.0 [3.7-4.3]	-
>6	3.4 [2.7-4.2]	1.4 [2.7]	4.2 [3.3-5.3]	-
**Current ART regimen has a PI**				
PI-based	16.1 [11.7-22.2]	36.3 [13.6-96.8]	2.1 [0.3-15.2]	18.6 [13.2-26.3]
Non-PI based	3.5 [2.4-2.7]	2.9 [2.6-3.3]	3.6 [3.5-3.8]	4.6 [3.5-5.9]
**Current facility**				
Clinic	3.5 [3.4-3.7]	2.9 [2.5-3.3]	3.6 [3.6-3.8]	4.9 [3.8-6.4]
Hospital	4.0 [3.5-4.6]	3.4 [2.2-5.1]	3.6 [3.1-4.2]	13.0 [9.1-18.4]

**Table 4:** Crude and Adjusted Incidence of developing Type 2 Diabetes mellitus in the study population and the associated factors

Parameter	cIRR [95%CI]	P-value	aIRR [95%CI]	P-value
**Sex**				
Male	1			
Female	0.77 [0.70-0.84]	<0.001	0.7 [0.6-0.8]	<0.001
**Current age (years)**	1.05 [1.04-1.06]	<0.001	1.054 [1.052- 1.058]	<0.001
**Marital status**				
Single	1			
Married	2.3 [2.2-2.6]	<0.001	1.3 [1.0-1.5]	0.026
Widowed/separated/Divorced	3.9 [3.3-4.6]	<0.001	1.3 [1.1-1.4]	<0.001
**BMI at ART Initiation**	1.06 [1.05-1.07]	<0.001	1.08 [1.07-1.08]	<0.001
**Duration on ART (years)** **ART Experience Status**	1.1 [1.09-1.12]	<0.001	1.04 [1.02-1.06]	<0.001
DTG Initiators	0.8 [0.6-1.0]	0.111	0.4 [0.3-0.6]	<0.001
DTG Transitioners	0.9 [0.3-1.4]	<0.001	0.4 [0.3-0.6]	<0.001
Non-DTG Users	1			
**Current ART regimen has a PI**				
PI-based	4.3 [3.1-5.9]	<0.001	1.9 [1.2-2.9]	0.005
Non-PI based	1			
**Current facility**				
Clinic	0.9 [0.8-1.0]	0.072	1.1 [0.9-1.3]	0.179
Hospital	1			

cIRR: crude incidence risk rate; aIRR: adjusted incidence risk rate;

* significance level at P<0.05;

** significance level at P<0.01.

*Model adjusted for age duration on ART, and baseline BMI*.

**Table 5:** Adjusted incidence risk rate for T2DM and risk factors by DTG Exposure status

Parameter	DTG Initiators Adjusted incidence risk rate (aIRR)	p-value	DTG Transitioners aIRR	p-value	Non-DTG Users aIRR	p-value
**Sex**						
Male	1		1		1	
Female	0.5 [0.4-0.7]	<0.001	0.7 [0.6-0.8]	<0.001	0.5 [0.3-0.9]	0.011
**Age (years)**	1.06 [1.05-1.07]	<0.001	1.053 [1.050- 1.056]	<0.001	1.03 [1.01- 1.06]	0.003
**Marital status**						
Single	1		1		1	
Married	1.6 [1.2-2.2]	0.003	1.2 [1.1-1.3]	0.002	1.8 [1.0-3.2]	0.046
Widowed/separated/Divorced	1.5 [0.8-2.8]	0.197	1.2 [1.0-1.5]	0.089	2.0 [0.6-6.2]	0.234
**BMI at ART Initiation**	1.09 [1.07-1.11]	<0.001	1.08 [1.07-1.09]	<0.001	1.06 [1.02- 1.09]	<0.001
**Duration on ART (years)** **Duration on DTG (years)**	1.0 [0.9-1.1]	0.817	1.05 [1.03-1.07]	<0.001	1.0 [0.9-1.2]	0.398
>=3	1				-	
4-6	0.6 [0.4-1.1]	0.093	1.1 [1.0-1.2]	0.089	-	
>6	0.5 [0.2-1.4]	0.201	1.0 [0.7-1.3]	0.839	-	
**Current ART regimen has a PI**						
PI-based	5.6 [2.4-13.2]	<0.001	0.7 [0.1-4.7]	0.684	1.7 [1.0-3.0]	0.047
Non-PI based	1		1		1	
**Current facility**						
Clinic	0.8 [0.5-1.3]	0.429	1.2 [1.0-1.5]	0.058	0.8 [0.5-1.4]	0.474
Hospital	1				1	

**ANNEX 1:**
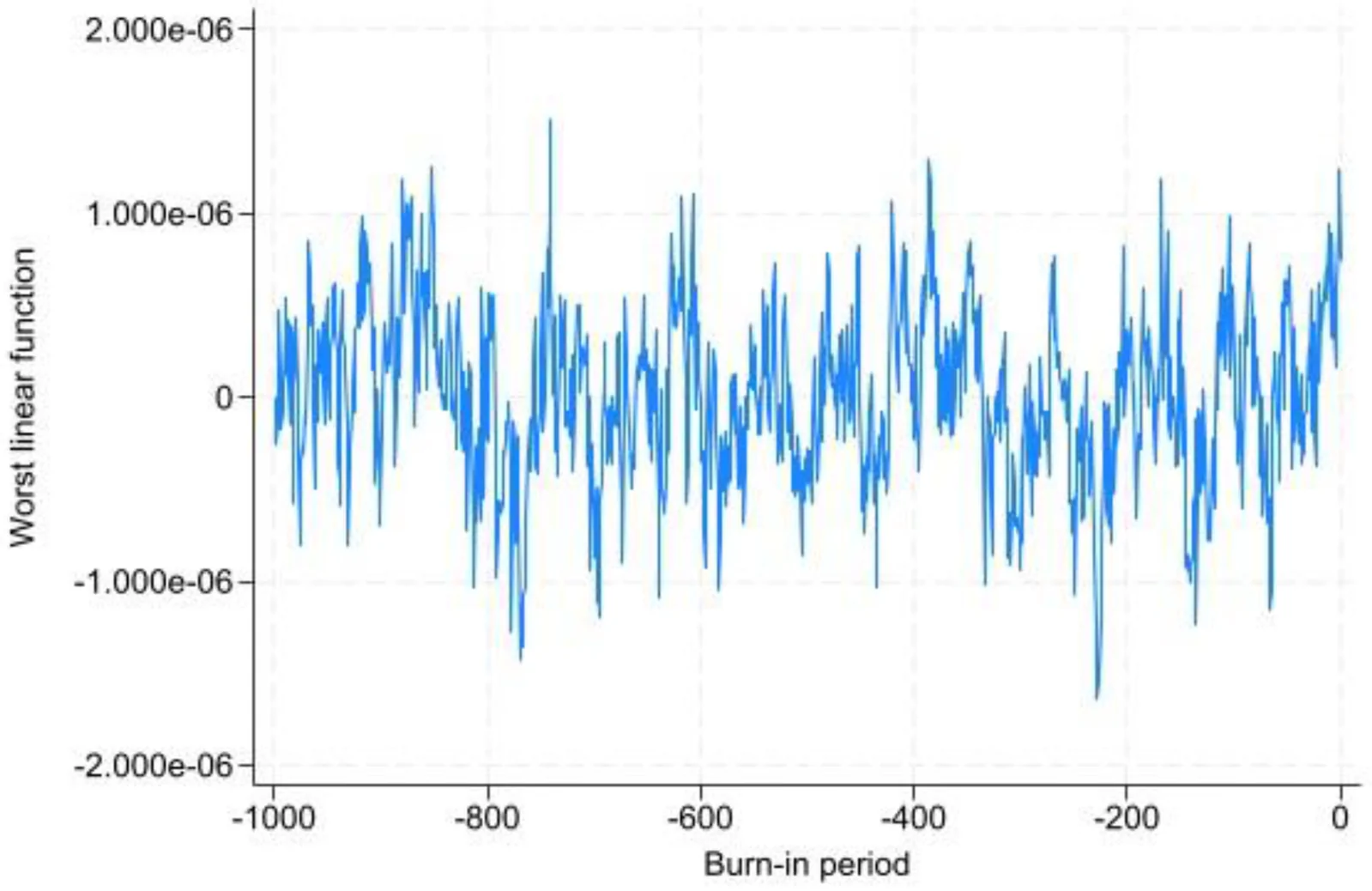
Autocorrelation

**ANNEX 2:**
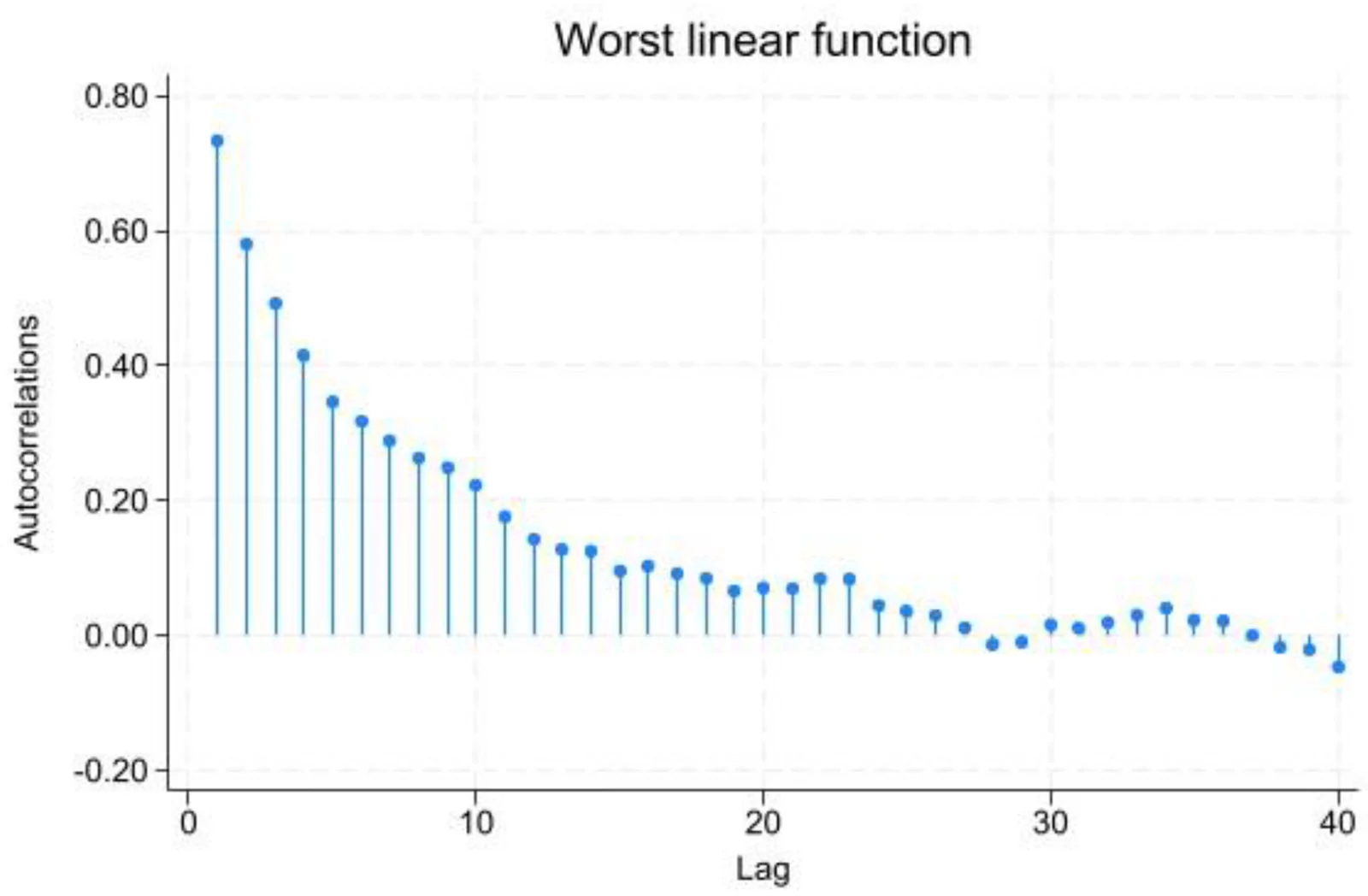
Worst Linear Function

## Data Availability

The data used for this study is available and will be shared by the corresponding author upon request.
